# Baseline Cognitive Control Predicts Weight Loss after Intervention in Middle and Older Adults

**DOI:** 10.1093/geroni/igaf122.3888

**Published:** 2025-12-31

**Authors:** Marrium Mansoor, Kristen Howard, Jenna Warnock, Theresa Libera, Jyoti Savla, Kevin Davy, Brenda Davy, Benjamin Katz

**Affiliations:** Virginia Tech, Blacksburg, Virginia, United States; Virginia Tech, Blacksburg, Virginia, United States; Virginia Polytechnic Institute and State University, Blacksburg, Virginia, United States; Virginia Tech, Blacksburg, Virginia, United States; Dept. of Human Development and Family Science, Blacksburg, Virginia, United States; Virginia Tech, Blacksburg, Virginia, United States; Virginia Tech, Blacksburg, Virginia, United States; Virginia Tech, Blacksburg, Virginia, United States

## Abstract

Cognitive processes, including attention and executive function (EF), are linked to multiple health behaviors including weight maintenance. In a 12-week clinical weight loss trial, 109 middle aged and older adults (50-70 years old) who had a BMI above 25 were randomized to control or lifestyle intervention (diet and physical activity) groups. Participants (76.4% female, 80% White) completed a set of cognitive tasks including the Attention Network Task (ANT) as well as physiological measurements at baseline and week 12. A mean weight change of -4.5 kgs (SD = 3.3) was observed across 12 weeks. A linear regression model was created with BMI change as the outcome with age, sex, race, education, intervention group, baseline BMI, and ANT performance as covariates. While experimental group was not associated with differences in BMI, reaction time (RT) on correct incongruent trials of the ANT significantly predicted BMI change (B= -.23, p=.033) suggesting that faster RTs on incongruent trials was linked to a larger decrease in BMI. Incongruent trials of the ANT are thought to reflect executive attention, the cognitive process that confers the ability to ignore irrelevant stimuli while focusing on the target stimulus or goal. This finding is consistent with other studies on EFs and weight loss and add to the literature by suggesting that executive attention may be an important cognitive process to consider in interventions aimed at helping individuals make lifestyle changes for weight loss health more generally. However, further research is needed to fully explore these links between EFs and weight maintenance.

